# Energy Return on Investment (EROI) for Forty Global Oilfields Using a Detailed Engineering-Based Model of Oil Production

**DOI:** 10.1371/journal.pone.0144141

**Published:** 2015-12-22

**Authors:** Adam R. Brandt, Yuchi Sun, Sharad Bharadwaj, David Livingston, Eugene Tan, Deborah Gordon

**Affiliations:** 1 Department of Energy Resources Engineering, Stanford University, 367 Panama St., Stanford, CA 94035, United States of America; 2 Carnegie Endowment for International Peace, 1779 Massachusetts Ave. NW, Washington, DC 20036, United States of America; Centro de Investigacion Cientifica y Educacion Superior de Ensenada, MEXICO

## Abstract

Studies of the energy return on investment (EROI) for oil production generally rely on aggregated statistics for large regions or countries. In order to better understand the drivers of the energy productivity of oil production, we use a novel approach that applies a detailed field-level engineering model of oil and gas production to estimate energy requirements of drilling, producing, processing, and transporting crude oil. We examine 40 global oilfields, utilizing detailed data for each field from hundreds of technical and scientific data sources. Resulting net energy return (NER) ratios for studied oil fields range from ≈2 to ≈100 MJ crude oil produced per MJ of total fuels consumed. External energy return (EER) ratios, which compare energy produced to energy consumed from external sources, exceed 1000:1 for fields that are largely self-sufficient. The lowest energy returns are found to come from thermally-enhanced oil recovery technologies. Results are generally insensitive to reasonable ranges of assumptions explored in sensitivity analysis. Fields with very large associated gas production are sensitive to assumptions about surface fluids processing due to the shifts in energy consumed under different gas treatment configurations. This model does not currently include energy invested in building oilfield capital equipment (e.g., drilling rigs), nor does it include other indirect energy uses such as labor or services.

## Introduction

The oil industry can be seen to have both economic and physical objectives. Economically, the oil industry tries to maximize the economic return from exploring, producing, and delivering to market a suite of energy-carrying substances from the Earth’s crust. From a physical perspective, the oil industry—along with other primary energy industries—is expected to supply energy to society in excess of that which it consumes. While this energetic goal is often not stated as an explicit objective, it represents the useful physical service provided by the oil industry to society.

Producing nearly 100 million barrels of petroleum per day is a non-trivial undertaking requiring a complex global industry. The oil industry consumes energy and capital in large amounts to find, extract, process and separate, transport, and refine oil into useful products. Some of these activities require the consumption of a significant fraction of the energy contained in the primary energy resource itself. The energetic “productivity” of the oil industry has been previously measured using various energy return ratios (ERRs) [1–4]. An ERR compares the amount of energy provided to society by a primary energy industry to the energy consumed by that industry. Thus, industries (or individual facilities) with high ERRs produce a large amount of energy relative to that which they consume. ERR definitions vary, with numerous ratios proposed since the 1970s. Common ERRs nclude EROI (energy return on investment) and the NER (net energy return) [1–3, 5]. At least 8 fundamentally different ERRs exist, and ERRs of different design can give different insights into the productivity of an oil resource [2, 3, 6].

The use of ERRs has emerged over decades as a tool for quantitative study of the effects of oil depletion and declining primary resource quality. Early scientists who studied oil depletion (such as Hubbert) focused on estimating future oil output and did not study trends in the energy returns from producing oil [7, 8]. Early work from an economic perspective examined trends in drilling depths and drilling costs per unit of oil produced [9]. Significant work in the 1980s examined trends in drilling efficacy in the US, generating time-series estimates of the yield of oil per unit of drilling effort applied (i.e., bbl of oil discovered per unit depth of oil well drilled) [10]. Later research used economic data sets on expenditures and consumption by the oil industry to examine trends in energetic returns from oil extraction [11, 12].

Recent work found that EROI for global oil and gas production peaked in 1999 and has since dropped [13], using global oil industry expenditures to estimate energy consumed. In that study, financial expenditure was multiplied by the energy intensity of the oil industry (or the economy in general for indirect expenditures) as computed for various regions. This approach is of particular interest because capital expenditure (capex) by the world’s largest international oil companies has been increasing by over 10% per year over the same post-2000 period [14].

Regional and national studies have been performed. One regional study tracked the EROI of the Norwegian oil industry, suggesting that energy returns from hydrocarbon (oil and gas) extraction peaked at approximately 60:1 in 1996 before registering a steady decline to around 40:1 by 2010 [15]. Most recently, researchers have studied energy returns from US oil extraction over nearly a century using (among other sources) historical statistics provided by the US Census of Mineral Industries to estimate direct and indirect energy consumption in the oil industry. They found unambiguous decreases in the energetic returns from producing oil [16].

Also, a suite of studies have looked at the energy returns implications of unconventional hydrocarbon resources. These studies have examined fundamentally different resources from conventional oil deposits, and examined the underlying reasons for differing energy returns from these resources. Studied resources include oil sands, oil shale, synthetic fuels, and hydraulically fractured oil and gas resources [17–23]. The general consensus of these studies is that unconventional hydrocarbon resources have lower energy returns than conventional oil resources (strongly variable by resource type). As this work focuses on conventional oil resources, we do not explore these studies further.

Lastly, energy returns can be studied using dynamic models of investment and production. For example, one study included oil energetic returns in a dynamic model of energy investment to understand the societal-scale impacts of declining oil EROI [24].

These prior studies are reasonably defined as “top down” studies. That is, they use macro-scale economic or physical consumption statistics for the oil industry in a given region or country to estimate the energy used directly and indirectly for producing oil. This perspective makes sense for a number of reasons. First, it gives an overall sense of the energetic efficacy of the industry at a society-wide scale (arguably the scale of greatest interest). Second, by using broad metrics of consumption with general sector- or economy-wide conversion factors, these studies are inclusive of many sources of energy consumption (e.g., system boundaries are defined broadly). Third, by leveraging historical statistical sources (often government datasets), long-term trends can be discerned.

However, these studies suffer from a drawback: the aggregation applied in reporting of industry-wide statistics destroys information about underlying physical drivers of energy returns. For example: does changing oil density (API gravity) affect energy returns, or does the depth of the average oil deposit in a region affect the energy returns from oil extraction? Does the rate of water co-production—which tends to increase as a resource depletes—impact the energetic efficacy of extraction? These questions are best answered using “bottom-up” engineering-based analysis of oilfield operations. Such analysis requires modeling the physical requirements of oil and gas production and processing.

Some tentative steps have been made in the direction of using engineering-based approaches to estimate energetic returns from the oil industry. First, Brandt explored the history of the California energy industry, using data from hundreds of oil fields with a simple physical model of drilling, oil lifting and steam generation [25]. More recently, Brandt et al. studied the energy returns from oil sands extraction using directly-reported energy consumption statistics provided by oil sands operators to the Canadian government [22]. A recent study examined well-to-wheels energy use and emissions for 30 global crude oils using two engineering-based life cycle analysis (LCA) models, one of which modeled oil production and one of which modeled crude oil refining [26]. Given the promise of increased understanding provided by a detailed engineering approach, this method is worth further exploration.

In this paper, we apply a detailed engineering-based model of oil production to assess ERRs from oil production for 40 global oil fields. These fields are located around the Earth and use a variety of extraction technologies. In order to estimate the energy used at each oilfield, we employ the Oil Production Greenhouse Gas Emissions Estimator (OPGEE), an open-source tool developed for assessing climate impacts from oil production [27, 28]. One goal of this study is to illustrate the use of OPGEE for computing energy returns, in order to facilitate other researchers in using this open-source tool for energy returns analysis. In addition, this study explores key data challenges, which are the chief impediment to further use of this method. The boundary of the system under analysis includes all oilfield operations up to the refinery inlet gate, and does not include energy for refining.

First, we describe the methods, datasets, and sensitivity cases included in this analysis. Second, we show results for each of the studied fields, emphasizing the variation in results that can arise from differing definitions of ERRs. Third, we explore the sensitivity of results to assumptions about oilfield technology embedded in the OPGEE model. Lastly, we conclude by discussing additional steps that could be taken to improve this method of analysis.

## Methods

This study uses the Oil Production Greenhouse Gas Emissions Estimator (OPGEE) to make estimates of the energy use in oilfields [27]. OPGEE estimates energy use and greenhouse gas emissions from “upstream” oil operations, defined as all activities between initial exploration and crude petroleum flowing through the refinery inlet gate. OPGEE is described in hundreds of pages of technical documentation and a number of peer reviewed studies [27, 28]. On-site energy use is modeled using the energy requirements of drilling, production, hydrocarbon processing, and transport. Offsite emissions associated with the provision of energy to the production site (e.g., upstream emissions associated with electricity production) are modeled using a broader life cycle emissions model based on the widely-used GREET fuel cycle model [29].

OPGEE requires the user to collect data on the operations at a particular oil field that is being modeled. Energy use at a given oilfield is dependent on the geological properties of the oilfield, properties of the produced crude, and technologies applied. Complex relationships exist between these parameters and the energy used in producing a crude oil. Example inputs that drive energy use at an oilfield include: depth of wells; production rate; water-oil-ratios (WOR); crude densities; reservoir pressure; injection of water, steam or other fluids into the reservoir; gas handling and gas cleanup; surface crude processing; and distance from the oilfield to the consuming location [30–32]. These data can be obtained from government bodies, technical datasets (e.g., from industry-affiliated groups like IHS), technical reports, academic papers, or oil industry conference proceedings. Of course, if the user of the OPGEE model works for an oil producer, directly collected information about each field can be obtained.

Because the global oil industry reports oilfield process data only sporadically, OPGEE uses default inputs and “smart default” relationships to estimate input parameters when all data are not available. Smart defaults leverage data that is known from a field in order to estimate a reasonable default value for an unknown parameter. For example, the water-oil-ratio (WOR, bbl of water produced per bbl of oil produced), is an important determinant of the energy cost of crude extraction. If the WOR of a field is not available in accessible public literature, OPGEE uses the field age to estimate the WOR using a relationship derived using historical data from dozens of large global oil fields [28].

Prior uncertainty analysis used the OPGEE model to explore the effects of limited oilfield data on resulting GHG and energy computations [30–32]. These studies found that a relatively small number of parameters were important determinants of oil field energy use and GHG emissions. These parameters include: steam injection rates for thermal oil recovery projects, WOR, depth of crude oil, and API gravity of the produced crude oil [30–32]. In addition, the rate at which associated gas is flared is known to be an important driver of emissions from oilfield operations, and if gas flaring is included in an ERR, results can change significantly.

OPGEE v1.1 Draft D [28] is used in this analysis. In all cases, the OPGEE “bulk assessment” feature was used which allows the analysis of multiple oilfields in a single run. The bulk assessment tool includes algorithmic methods for assessment of crude oils as described in OPGEE technical documentation [28]. Because the fields are assessed as a group, further customization of the modeling of each field (beyond inputs customizable in the bulk assessment sheet) is not performed.

We generated a global range of crude oil operations by creating a dataset with 40 global oil fields [26]. This dataset allows for input of up to 60 parameters for each oil field, as allowed in the OPGEE bulk assessment tool. These data were collected as part of the Oil Climate Index project, lead by the Carnegie Endowment for International Peace [26]. These data were collected from scientific and professional literature (e.g., Society of Petroleum Engineers journals and conferences), project technical documentation (e.g., operator compliance documents or development plans), and regulatory sources (e.g., information reported to oil and gas regulatory bodies). Where data are not available for a given field, the bulk assessment tool fills in the appropriate value from OPGEE default and “smart-default” relationships. Over 180 sources were cited [26]. See Supporting Information (S1 Worksheet) for citations, assumptions, and resulting input data for all 40 oilfields. Despite the wide-ranging collection of data for these oilfields, data quality remains a concern (see discussion below).

In collecting this comprehensive dataset, data availability was found to vary by parameter: some information was available for all or nearly all fields (e.g., field depth, oil production rate), while other parameters were generally unavailable, even when searching across dozens of publications on a given oil field (e.g., surface processing design or reservoir productivity index). Some of these data are unavailable due to lack of government reporting requirements, while others may be commercially sensitive and therefore not be reported. Overall, the dataset used represents a robust effort at data collection for a large group of fields, but uncertainties and problems remain. For some fields, data are not available from trustworthy or recent sources. Depletion-linked parameters such as steam-oil-ratio or water-oil-ratio may can change significantly over time, rendering older data less useful. We explore the impacts of uncertainty in these data inputs using sensitivity analysis below.

**Fig 1:**
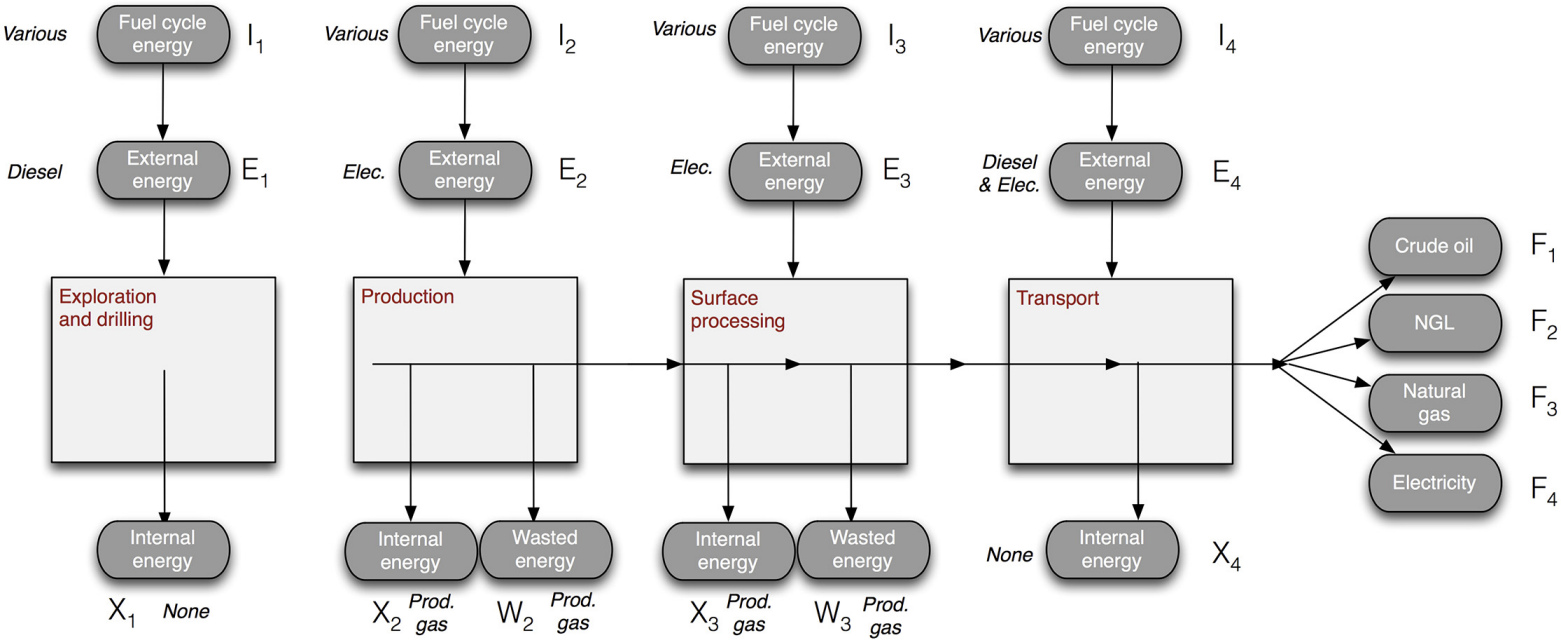
System diagram with flow labels as used in equations. Consistent assumptions about type of energy across fields allows easier characterization of upstream emissions.

After assessment using OPGEE, relevant energy flows are exported from OPGEE results sheets. These flows are classified as shown in Fig 1 below.

While quantities of energy consumed in each field differ based on model results, for consistency all fields are assumed to consume consistent forms of energy for a given process or requirement. In all 40 fields we therefore assume that:

- Exploration and drilling are powered with diesel engines.
- Production operations are powered with electric drive when pumping, mixing, compression, or agitation are required (e.g., downhole pumps, reinjection compressors). Any thermal requirements for production operations are met with on-site produced hydrocarbon gas, supplemented with external natural gas where on-site production is insufficient (uncommon). On-shore operations are assumed to purchase electricity from the grid, while offshore operations produce electricity on site using simple cycle gas turbines fueled with produced hydrocarbon gas.
- Processing operations are powered with on-site produced natural gas where thermal requirements dominate (e.g., heater/treaters or heated stabilization separators). For electricity needs in processing, on-shore operations procure electricity from the grid, while offshore operations produce electricity on site using simple cycle gas turbines fueled with produced gas.
- Transport energy is assumed provided by petroleum fuels (either heavy fuel oil or diesel) for tankers, barges, and rail. Pipelines are assumed powered by electricity. All transport of crude is modeled using the distance from each field to reach a common destination (Houston, TX, USA).
- All electricity is provided by the default OPGEE grid mix (GREET model of US typical grid, 46% coal, 23% natural gas, 20% nuclear, etc.)

Note that these assumptions may not strictly hold for any given field. For example, some onshore fields will not purchase electricity but generate it onsite. Also, many of these crudes may not in fact be shipped to Houston in practice. However, the use of consistent assumptions enables more intercomparison between fields and improves our understanding differences in resulting ERRs. We expect the normalization benefits of consistent assumptions to outweigh the loss of granularity in modeling each field.

Upstream or “fuel cycle” consumption is the added energy required to supply any external energy to the oilfield. For example, each MJ of diesel consumed at an oilfield requires additional consumption of 0.23 MJ of energy for its production, refining, and transport, while each MJ of electricity used on site requires consumption of 2.41 MJ of fuel-cycle energy during its generation (for the particular grid mix modeled here). The fuel cycle calculations in OPGEE are taken from the GREET model (version GREET.1.2013) [33].

We follow the method of Brandt et al. [2, 3] to define ERRs for studied oilfields. Brandt et al. listed 8 different ERRs that were previously defined in decades of literature on ERRs. While ERRs generally represent a ratio between energy “outputs” and energy “inputs”, each ratio can differ in what inputs are considered, what outputs are considered, how internal consumption is counted. Murphy et al. [1] define a number of standard EROI formulations that contain many of these historical definitions.

First, we can differentiate between different sources of energy inputs (i.e., denominator of ERRs). *Internal* energy inputs are provided by diverting some portion of the produced energy resource to power on-site operations. *External* energy inputs are provided from an energy carrier that is procured offsite from the operation under study. A ratio that includes all energy inputs (internal and external) is called a net energy ratio (NER). In contrast, an external energy ratio (EER) is computed considering only offsite energy inputs.

In all cases, diesel fuel is considered an external input. Although any oilfield being studied could be used to produce diesel, the diesel fuel used in a field generally comes from another oilfield by way of a refinery rather than an on-site process. Electricity is always assumed to be an external input except in cases of offshore production, where on-platform electricity generation is common. Because the OPGEE model cannot currently estimate the energy used in constructing oilfield equipment (e.g., wells, offshore platforms), this form of “embodied” external energy input is not included in the analysis.

In addition to differentiation between input energy quantification (between NERs and EERs as defined above), we also differentiate ratios based on which output energy streams are included in the numerator of the ERR. We compute ratios which count only the produced crude oil, as well as ratios that include all energy sources co-produced with the oil (such as natural gas or exported excess cogenerated power). Recall that this represents an analysis of extraction energy and does not include refining energy use.

Using the flow enumeration scheme of Fig 1, we can define the following flow types: flows *F* are the flows of final energy produced for use in other sectors; flows *X* are internal energy resources consumed on site for powering oilfield equipment; flows *E* are external energy inputs (e.g., electricity purchased from the grid); and flows *I* are the indirect inputs in the rest of the economy required to supply flows *E*. For example, to supply one unit of natural gas to the oilfield, 0.07 units of energy are consumed elsewhere in the economy to procure and supply that energy from another gas field. Use of on-site energy sources does not require these indirect inputs. Lastly, flows *W* are wasted energy products, typically flared gas, but also operational venting or other product loss. Again, note that embodied indirect inputs (e.g., energy use for steel and cement) are not currently included in the OPGEE model. Using definitions from previous work [2] our four ERRs are defined as:

$$NER_{oil}=\frac{F_{1}}{\sum_{i=1}^{n}\;X_{i}+\sum_{i=1}^{n}\;E_{i}+\sum_{i=1}^{n}\;I_{i}}$$ (1)

$$NER_{tot}=\frac{\sum_{j=1}^{p}\;F_{j}}{\sum_{i=1}^{n}\;X_{i}+\sum_{i=1}^{n}\;E_{i}+\sum_{i=1}^{n}\;I_{i}}$$ (2)

$$EER_{oil}=\frac{F_{1}}{\sum_{i=1}^{n}\;E_{i}+\sum_{i=1}^{n}\;I_{i}}$$ (3)

$$EER_{tot}=\frac{\sum_{j=1}^{p}\;F_{j}}{\sum_{i=1}^{n}\;E_{i}+\sum_{i=1}^{n}\;I_{i}}$$ (4)

In each case above, *n* represents the number of process stages being analyzed and *p* represents the number of energy products produced, with counter indices *i* and *j* respectively. In nearly all fields, *n* = 4, as shown in Fig 1. In one case—the Hamaca field of Venezuela—upgrading of extra-heavy petroleum occurs before long-distance transport of the crude, adding upgrading as a fifth process stage. In all cases, up to four output products *p* are possible: crude petroleum, natural gas liquids (NGLs), natural gas, and electricity.

A note about terminology is in order. Energy that is used on site (within the geographic boundary of the oilfield) is always included as “direct energy use”, which can be made up of both “internal” energy (energy produced from the field itself) and “external” energy (energy procured from offsite but used onsite). In contrast, “indirect” energy use occurs offsite in order to provide the external energy imported by the project. A few examples will suffice to explain further. Produced natural gas that is burned on site to fuel heated separation equipment is a direct and internal energy use. Diesel fuel that is purchased by the exploration company is a directly consumed external energy input. To produce this diesel fuel from another oilfield and get it to the drilling rig requires some indirect energy consumption in a refinery and other process stages.

*NER*_*oil*_ and *NER*_*tot*_ are similar to EROI metrics developed in other studies for oil-only energy returns and oil and gas energy returns, respectively. Because these ratios all include only extraction but not energy used during processing or end use, they correspond to the Murphy et al. “Boundary 1” for energy outputs [1]. These ratios include both direct energy use and indirect energy use, they are similar to the Murphy et al. “Boundary 2” for energy inputs. Therefore, these ratios are most similar to EROI_2,1_, otherwise known as the “standard EROI” or EROI_*std*_. However, note that this analysis does not include energy embodied in materials consumed, because of the lack of coverage of embodied emissions in OPGEE. Therefore, our results will be “optimistic” approximations of EROI_*std*_ from these oilfields, and should not be considered to be direct implementation of the EROI_*std*_ methodology. The EER metric is classified by Murphy et al. as an alternative to EROI, and so has no close analogue in the EROI literature [1].

In keeping with OPGEE default settings, all thermal energy sources are measured on a lower heating value (LHV) basis in both numerator and denominator of ratios. Depending on the relative importance of different products (e.g., NG vs. oil output) and different inputs (e.g., natural gas vs. electricity), the NER as measured on a higher heating value (HHV) basis could differ slightly due to different HHV/LHV ratios of different primary fuels (i.e., fuels containing more hydrogen will emit more water vapor and have a larger divergence between LHV and HHV measures of energy content). All electricity quantities (inputs or outputs) are converted to primary energy basis using default OPGEE fuel cycle consumption factors (weighted efficiency ≈ 29% from well-to-wall-socket, including upstream fuel consumption and transport/distribution losses).

For ease of replication by other researchers, Table 1 shows the locations in the OPGEE model where data were extracted for computing each flow in Fig 1.

**Table 1:** Calculation methods for flows used in ERRs.

Flow	Type	Unit	Sheet	Cell	Notes
E1	Diesel	mmBtu/d	EC^a^	H20	
I1	Upstream diesel	mmBtu/d	FC^b^	D57	E1 × diesel fuel cycle factor of 0.23 Btu/Btu
E2	Electricity	mmBtu/d	EC	H28	Electricity for N_2_ separation
X2	Natural gas	mmBtu/d	EC	Sum H23–27; 29–32	
I2	Upstream elec.	mmBtu/d	FC	D58	E2 × electricity fuel cycle factor of 2.41 Btu/Btu.
E3	Electricity	mmBtu/d	EC	Sum H40–41; 45; 50–53	Sum of all electricity uses in surface processing
X3	Natural gas	mmBtu/d	EC	Sum H36–37; 42; 47–48	
I3	Upstream elec.	mmBtu/d	FC	D58	E3 × electricity fuel cycle factor of 2.41 Btu/Btu.
E4	Diesel and elec.	mmBtu/mmBtu	T^c^	M98–102	Diesel and electric consumption summed separately
I4	Upstream diesel and elec.	mmBtu/mmBtu	FC	D57, 58	Diesel and electric fuel cycle factors applied separately
X5	Upgrader residues	mmBtu/mmBtu	BAR^d^	H143	
F1	Oil	mmBtu/d	BAR	H22	Multiplied volume bbl/d in BAR by energy density of crude in “Fuel Specs” sheet M14
F2	NGL	mmBtu/d	EC	E79	If EC E79 < 0, export NGLs. Exports = −E79
F3	Natural gas	mmBtu/d	EC	E78	If EC E78 < 0, export natural gas. Exports = −E78
F4	Electricity	mmBtu/d	EC	E81	If EC E81 < 0, export electricity. Exports = −E81. Use electricity fuel cycle factor of 2.41 Btu/Btu.

^a^ - EC = OPGEE “Energy Consumption” sheet

^b^ - FC = OPGEE “Fuel Cycle” sheet

^c^ - T = OPGEE “Transport” sheet

^d^ - BAR = OPGEE “Bulk Assessment—Results” sheet

In order to represent the environmental impacts of oil and gas production better, we include a second version of the *NER*_*tot*_ metric: one in which flaring energy use is included as a consumed energy input. Flaring represents a wasted energy stream that is expended in producing the oil, at least the way certain fields operate at present. Because of these operational choices, flaring represents an energy cost of operations, although one that could be avoided through better management of associated gas or through regulatory requirements for flaring reduction. *NER*_*tot*, *flare*_ is defined as follows:

$$NER_{tot,flare}=\frac{\sum_{j=1}^{p}\;F_{j}}{\sum_{i=1}^{n}\;X_{i}+\sum_{i=1}^{n}\;E_{i}+\sum_{i=1}^{n}\;I_{i}+\sum_{i=1}^{n}\;W_{i}}$$ (5)

Note that this equation differs from *NER*_*tot*_ through its inclusion of wasted energy (*W*_*i*_) per process stage in the denominator.

In order to explore drivers of ERR values, we create groups of fields by binning them using characteristics that were previously noted to result in higher GHG emissions [30–32, 34–36]. We can then examine trends in the ERRs of these groupings as compared to the total population of fields. We create the following groupings of fields (not mutually exclusive):

- High WOR: WOR > 10 bbl water/bbl oil
- Deep: deeper than 10,000 feet below surface or seafloor
- Ultra-deep: deeper than 15,000 feet below surface or seafloor
- Old: age of field greater than 40 years
- Heavy oil: API gravity < 22.5°API
- Ultra-Heavy oil: API gravity < 15°API
- Thermal EOR: thermal recovery option is turned on in OPGEE settings

Sensitivity analysis is performed to explore the impact of engineering assumptions on the values of ERRs. Note that detailed uncertainty analysis was performed in three previous papers focused exclusively on uncertainty, reproducibility and data requirements for the OPGEE model [30–32]. In those papers, it was found that important factors driving energy use and emissions include lifting energy (affected by water-oil-ratio and efficiency of pumps), thermal energy for steam injection (affected by temperatures and generator efficiencies), and the processing configuration. Therefore, we explore the effect of model assumptions in these critical areas below.

First, we explore the most energy intensive fields, those that practice thermal EOR. Modeling thermal EOR requires a number of assumptions about steam generation technology. Two cases, low-intensity and high-intensity thermal EOR, are defined in Table 2. These sensitivity cases are applied only to the three fields in our dataset that apply thermal EOR. Table 2 also outlines a number of other sensitivity cases which are applied to all 40 fields, exploring variation in drilling energy intensity, fluid lifting energy intensity, and surface processing practices.

**Table 2:** Sensitivity case definitions.

Setting	Default	Low sens.	High sens.	V high sens.	Units	Notes
Thermal EOR sensitivity						
- Inlet water temperature	40	140	40	-	°F	
- Excess air ratio	1.2	1.15	1.25	-	O2/O2 stoich.	
- OTSG exhaust T	350	325	400	-	°F	a
- OTSG shell loss	0.04	0.03	0.05	-	MJ/MJ	
- Cogen. GT efficiency	30.9	35.8	26.2	-	%	b
- Cogen. HRSG exhaust T	350	325	400	-	°F	c
- Cogen. HRSG shell loss	0.05	0.03	0.075	-	MJ/MJ	
Lifting energy sensitivity						
- Friction factor	0.02	0.01	0.03	-	[-]	
- Pump efficiency	65	70	60	-	%	d
- Compressor efficiency	75	80	70	-	%	e
Drilling energy sensitivity						
- Drilling energy setting	Low	Low	High	High	[-]	
- Energy multiplier	1	0.75	1	1.5	[-]	f
Processing configuration sensitivity						
- Heater/treater	0	0	1	-	Y/N	
- Stabilizer	1	0	1	-	Y/N	
- Acid gas removal	1	0	1	-	Y/N	
- Gas dehydration	1	0	1	-	Y/N	
- Demethanizer	1	0	1	-	Y/N	

*a* - OTSG = once-through steam generator

*b* - GT = Gas turbine in co-generation system. These settings represent turbines C, D, and B, respectively in OPGEE cogeneration settings (C = default)

*c* - HRSG = heat-recovery-steam-generator

*d* - This pump efficiency is applied to both downhole lifting pumps and water reinjection pumps

*e* - This compressor efficiency is applied to both gas lifting compressor and gas reinjection/gas flooding compressor

*f* - This is a pre-multiplier on the default exponential relationship selected in the drilling energy setting.

## Results

**Fig 2:**
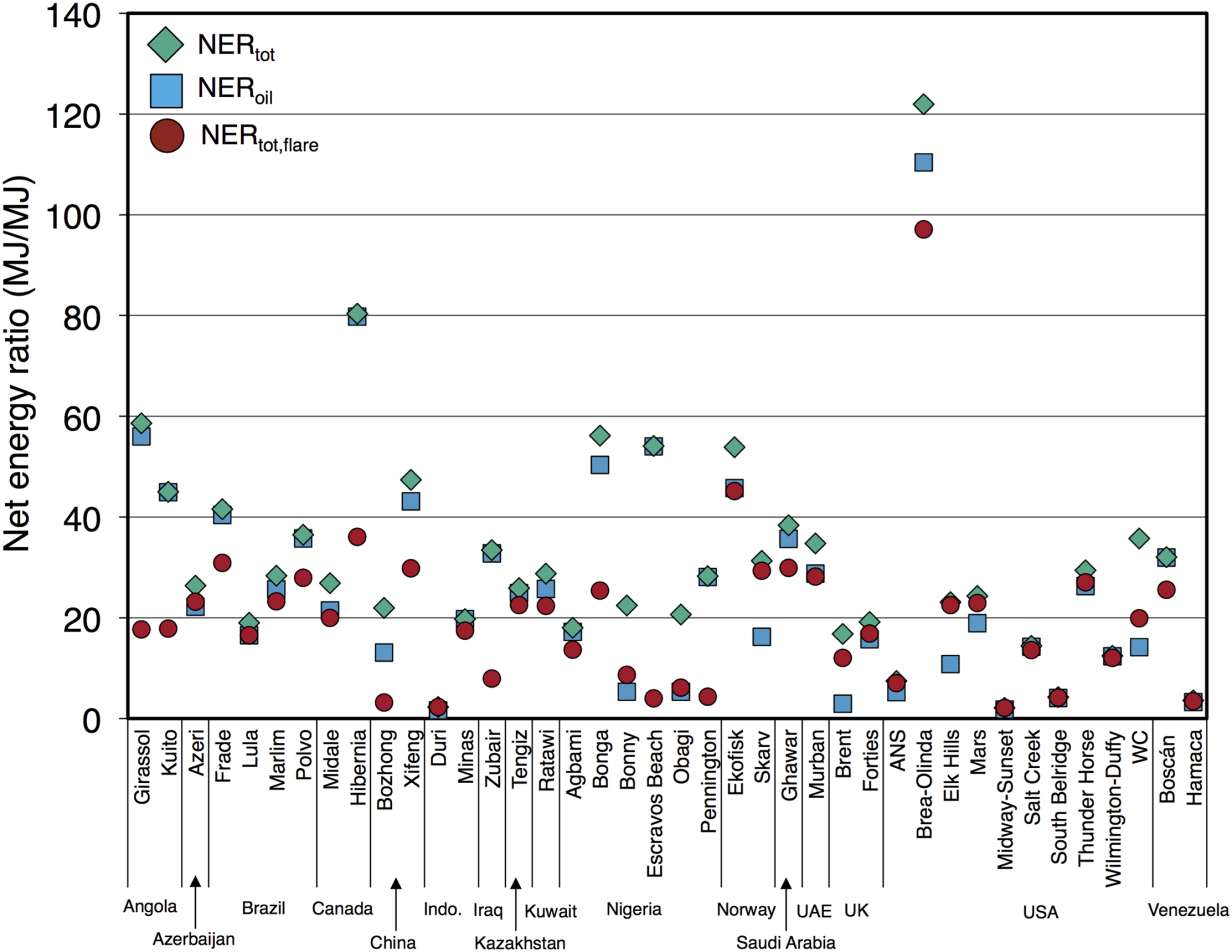
Total net energy ratio *NER*_*tot*_ and oil-specific net energy ratio *NER*_*oil*_ for studied global oil fields. *NER*_*tot*_ is always greater than or equal to *NER*_*oil*_. *NER*_*tot*, *flare*_ adds in energy content of flared gases as consumed energy (see discussion in main text).

Fig 2 shows that *NER*_*oil*_ for 40 global oil fields varies from less than 2 MJ/MJ in the Duri and Midway-Sunset fields to over 100 MJ/MJ in the Brea-Olinda field. The total NER for all products, *NER*_*tot*_, is by definition greater than or equal to the *NER*_*oil*_. *NER*_*tot*_ is virtually identical to *NER*_*oil*_ for fields with small amounts of NG, NGL, or electricity co-production. In some cases, the *NER*_*tot*_ is much higher than *NER*_*oil*_. For example, the Brent field is currently being managed as a gas field: the reservoir pressure is being purposely lowered to produce large quantities of gas, and the gas-oil-ratio (GOR) is nearly 30,000 scf/bbl. Therefore, measuring Brent energy returns on an oil-only basis results in a greatly diminished NER. The NER including flaring energy use (*NER*_*tot*, *flare*_) is significantly lower than the *NER*_*tot*_ in a number of cases (e.g., Nigeria Pennington).

Table 3 shows summary statistics for *NER*_*tot*_ for the sub-groups of fields defined above, as well as for all 40 fields. The production-weighted mean *NER*_*tot*_ for all 40 fields is 32.5 MJ/MJ. High WOR and deep fields tend to have lower *NER*_*tot*_ values due to lifting energy requirements (production-weighted mean values of 12.3 and 29.7 MJ/MJ, respectively). WOR is more impactful to *NER*_*tot*_ than depth, because greater depth is partially offset by higher default reservoir pressure, helping to offset lifting energy requirements. Also, WOR varies by a factor of 600 between highest and lowest WOR, a much larger range of variation than between the shallowest and deepest fields (see SI datasheet for input WORs for each field). Heavy and ultra-heavy oil have increasingly low NERs, due to greater importance of thermal recovery in these operations (production-weighted mean *NER*_*tot*_ of 17.7 and 10.6 MJ/MJ, respectively). The lowest *NER*_*tot*_ values were observed in the thermal recovery sub-group. These fields have a production-weighted mean *NER*_*tot*_ of only 2.8 MJ/MJ. Age is not a significant driver of *NER*_*tot*_, perhaps because the significant old oilfields in this study are reasonably productive (major global oilfields) rather than fields that are highly water intensive (i.e., not significant overlap between high WOR and old field categories). This result differs from the case where the same fields are examined over time as they deplete and water production increases [25], which was found to result in lower *NER*_*tot*_ over time. It is of general interest that across many “categories” of oilfields, the weighted mean NERs varied within a reasonably tight range of 10–35 MJ/MJ, excepting thermal EOR.

**Table 3:** Groups of fields and resulting *NER*_*tot*_ measures.

	N fields	Mean	Weight. mean	Median	SD	Notes
All fields	40	31.2	32.5	27.6	22.3	
High WOR	6	16.3	12.3	15.6	8	a
Deep	12	30.2	29.7	27.1	16.5	b
Ultra-deep	3	24.3	22.3	24.4	5.2	c
Old	15	25.2	35.9	26.9	16.6	d
Heavy oil	10	20.8	17.7	20.4	17.6	e
Ultra-heavy oil	3	12.5	10.6	3.3	16.9	f
Thermal EOR	3	3	2.8	2.5	1.2	g

*a*—High WOR is defined as water production above 10 bbl water/bbl oil

*b*—Deep is defined as deeper than 10,000 feet below surface (or below seafloor)

*c*—Ultra-deep is defined as deeper than 15,000 feet below surface (or below seafloor)

*d*—Old is defined as an age greater than 40 years

*e*—Heavy oil is defined as API gravity <22.5°API

*f*—Ultra-Heavy oil is defined as API gravity <15°API

*g*—Thermal EOR is defined as having the thermal recovery option turned on in OPGEE settings

**Fig 3:**
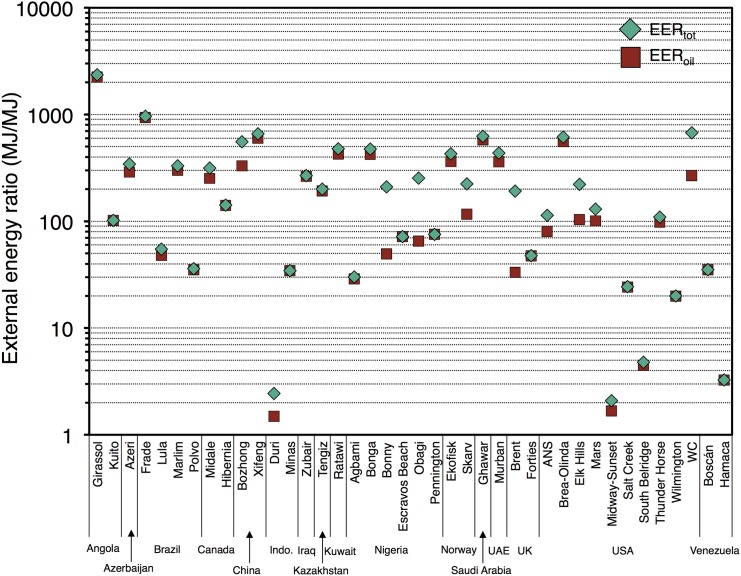
Total external energy ratio *EER*_*tot*_ and oil-specific external energy ratio *EER*_*oil*_ for studied global oil fields. Note logarithmic scale due to very wide variation in *EER* values.

Fig 3 shows the external energy ratios (*EER*_*tot*_ and *EER*_*oil*_) for our 40 global oilfields on a logarithmic scale. By definition, EERs are always greater than or equal to the equivalent NERs. Because of the assumption of preferential internal use of associated gas before import of natural gas for thermal energy requirements, EERs tend to be significantly higher than NERs in most cases. Flaring does not affect EERs due to gas consumption being an internal product (i.e., companies do not purchase external natural gas only to flare it).

*EER*_*tot*_ in studied fields can reach over 1,000 MJ/MJ. Very high *EER*_*tot*_ is associated with offshore operations where electricity is assumed produced on site, meaning that offshore fields are largely self-sufficient and require little external energy except for drilling and long-distance transport of crude. In some cases, especially fields practicing thermal EOR, each *EER*_*tot*_ is very similar to the equivalent *NER*_*tot*_. This is due to the fact that these fields produce small amounts of associated gas and have very large thermal energy inputs for steam generation, requiring imports of 20–40% of the energy content of the crude oil as NG. This factor affects the NERs and EERs in a similar fashion.

The self-fueled nature of some offshore operations, while resulting in high EERs in our model, should not be assumed to represent a physically or financially optimal outcome. For example, offshore operations with larger quantities of associated gas than can be used for on-site energy generation often flare the excess gas in the absence of easy access to markets or other credible prospective consumers of the gas (see Nigerian cases above). Moreover, offshore operations may not retain consistently high EERs in the future. There has been growing interest in recent years in the prospect of powering offshore operations with on-shore generated electricity by way of high voltage power lines. The first power-from-shore oilfield link was implemented in 2003 at the Abu Safah development off of Saudi Arabia, and two Norwegian platforms (Troll and Valhall) have since followed. Norway is now considering a 100 MW high voltage direct current submarine cable that will power future development of the Johan Sverdrup field from 2019 onwards [37].

It is not possible to draw clear environmental conclusions from EER metrics. A large EER does not imply that environmental impacts are small. A resource may burn large amounts of co-produced natural gas and have a high EER, yet also have high GHG impacts. In this respect, NER is a better metric for overall energy intensity, which will better correlate with environmental consequences.

**Fig 4:**
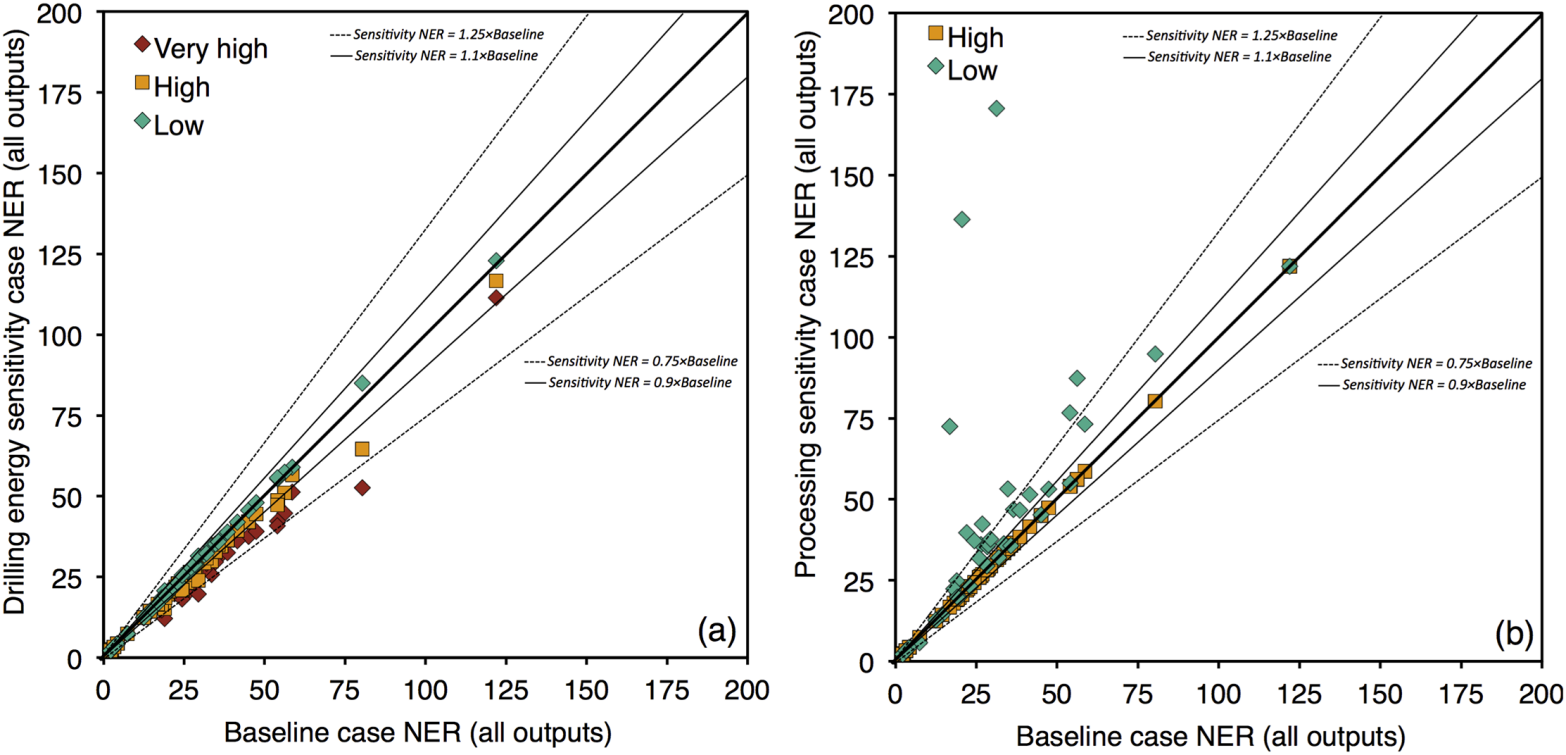
Sensitivity of all studied fields to two varied parameters. (a) drilling energy sensitivity case and (b) processing configuration sensitivity cases (see case definitions in text).

We next discuss the general sensitivity analysis results. In most cases, results were not sensitive to the ranges of assumptions explored above in Table 2. For example, results are generally insensitive to the lifting energy assumptions explored in Table 2, with variation in *NER*_*tot*_ in all fields less than 10%. *NER*_*tot*_ results are also (in most cases) insensitive to drilling requirements (see Fig 4a). This is in line with previous results from Norway, where drilling was a minor contributor to energy returns values [15]. Fig 4a shows that in most cases, drilling energy sensitivity cases result in variation of less than 25% in the values of *NER*_*tot*_. One exception is the case of Hibernia in offshore Canada, which has a large number of relatively deep wells for its level of productivity.

In contrast, very large variability in *NER*_*tot*_ is observed upon changing the surface fluid processing design in some fields (see Fig 4b). Four different fields realize an *NER*_*tot*_ increase by greater than 4 times the base value when the gas processing intensity is set to low processing intensity (e.g., turn off acid gas removal, demethanizer, and dehydrator). These fields include Bonny, with a 13.4 times increase in *NER*_*tot*_, Obagi with a 6.6 times increase, Skarv with a 5.5 times increase, and Brent with a 4.3 times increase. Three of these four fields have GORs near or in excess of 20,000 scf/bbl (see SI worksheet for data inputs), implying large energy requirements for gas processing in the default configuration. These variations should be considered part of the uncertainty associated with modeling a field where the specific processing configuration is not known by the modeler. Because required product specifications and the quality of the underlying resource are given quantities, the choice of processing configuration is not one set completely by free operator choice (e.g., operators cannot generally choose to avoid desulfurizing sour gas to avoid the energy cost of acid gas removal, as the resulting gas would not meet quality specifications).

**Fig 5:**
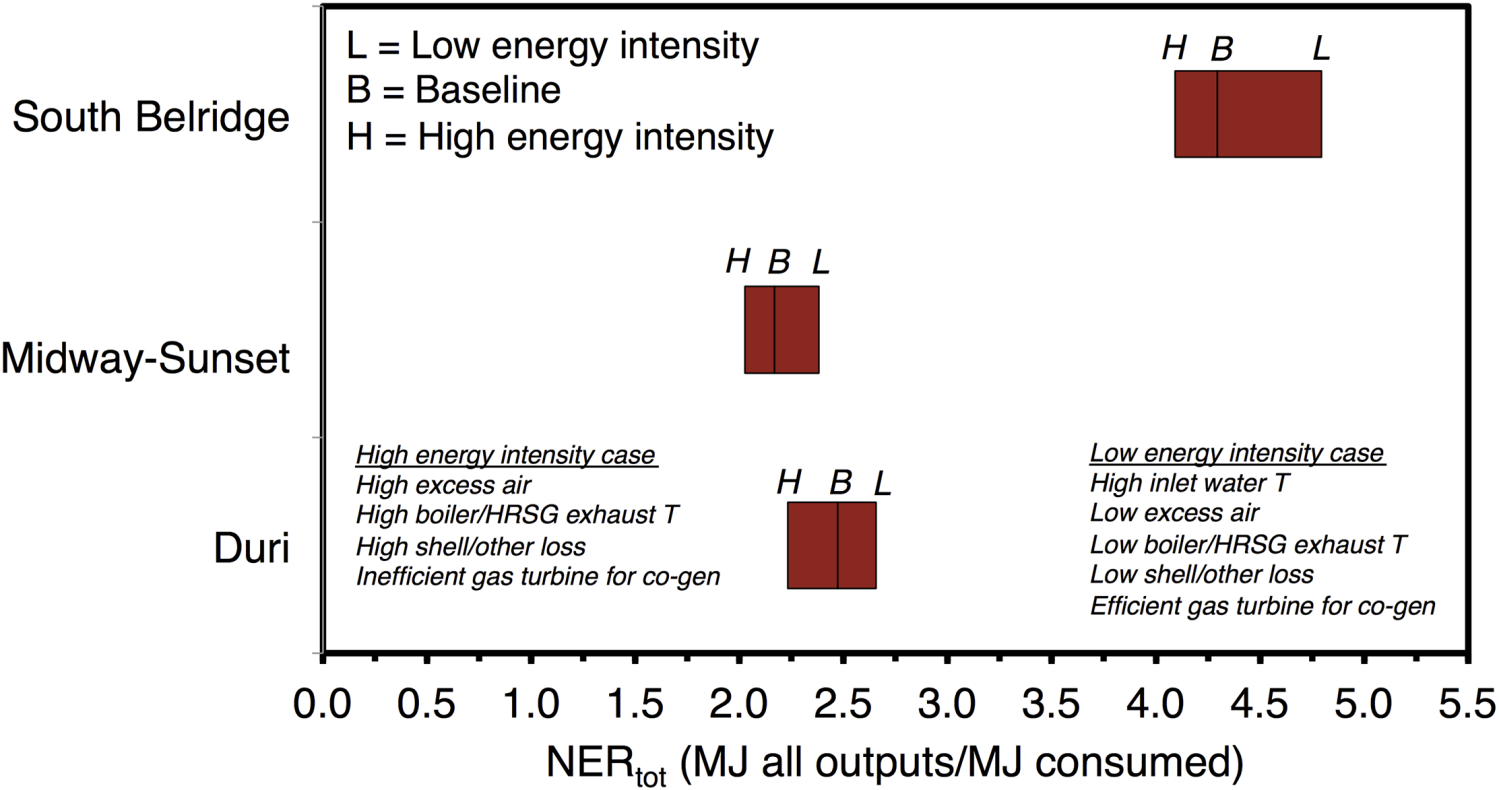
Sensitivity of low-NER thermal oil recovery fields to assumptions about steam generation efficiencies and configurations.

Thermal oil recovery NERs are quite low, so it is worthwhile to examine the sensitivity of results to various assumptions. We find that ERR results are only mildly sensitive to assumptions made in modeling thermal EOR. Fig 5 shows the variability in thermal EOR field values for *NER*_*tot*_, adjusting model settings as defined in Table 2. Although the range of variability in input parameters may seem small (e.g., 1.15–1.25 mol/mol excess air ratio), these settings represent the range of reasonable operating ranges presented in the engineering literature. For example, while steam exhaust temperatures could in theory be much higher than those explored, but no sensible operator would voluntarily choose such high exhaust temperatures because that would result in significant energy waste and economic costs. The most important sensitivity parameter in these studies is the inlet water temperature. In the low intensity case, inlet water temperature to the boiler increases to 140°F. This representative of a system where large amounts of warm produced water is recycled into steam boilers.

## Discussion

The net energy return ratios (NERs) examined in this study for global oilfields range from approximately 2:1 to 100:1, with a production-weighted mean of 33:1. These results are most sensitive to changes when the gas processing configuration is unknown (as discussed above) or where the properties of injected steam are unknown. The implications of these relative magnitudes of energy returns are robust to explored uncertainties. A resource with an small energy return ratio may face challenges in scaling output, will consume large amounts of energy for production, and likely cause large environmental and climate impacts per unit of energy produced. This range of observed NER ratios is within the range suggested in prior literature. This agreement is a sign of convergence between methodologies: while the method used here is much more specific than prior methods, in that it models the engineering processes in oil extraction in greater detail, it provides similar order of magnitude results as methods used elsewhere.

However, direct comparison between these results and prior work is challenging. This paper does not include as wide of a system boundary as used in other studies (e.g., [16]). For example, that work uses financial information to impute widely distributed indirect energy uses, while our model only includes indirect energy consumed to produce the energy inputs used in the oilfields. Therefore, our figures are more likely to be overestimates of EROI or NER than underestimates of these ratios. Also, we do not include an allocation of dry holes or other wasted expenditure within these operating projects (e.g., for each discovered oilfield, some number of dry holes are always drilled). For these reasons, caution is to be used in making very specific or detailed comparisons between these results and prior results.

Importantly, applying the assumption that produced natural gas powers processing operations, the resulting EERs are quite high. For a number of offshore fields that require self-sufficiency in terms of energy supply, the amount of energy supplied to society is in some cases thousands of times larger than the amount of energy consumed from the rest of society. This large multiplicative factor has important implications for the ability of energy systems to grow over time and supply net energy to the industrial ecosystem.

Future work should focus on obtaining information for additional global oilfields in order to better sample the range and central tendency of ERRs for global oilfields. A more sophisticated uncertainty analysis would allow examination of multiple uncertainties simultaneously, rather than the simple single-factor sensitivity analysis presented here. A great challenge, well-noted in decades of literature, is the poor availability of data from some oil producing regions. Some of the data in our large dataset are of questionable quality and can likely only be considered marginally better than ignorance or reliance on default values. For example, very little data is available on the prolific oilfields of OPEC, which supply a large fraction of global oil output. The effect of data availability on the accuracy of OPGEE results is well explored in previous work [30, 31]. In these papers we explore the amount of data required to obtain a certain accuracy threshold, and determined which data are most important for reducing uncertainty, and the conclusions drawn there apply here as well when computing ERRs.

Also, a broadening of system boundaries to include embodied energy in oilfield capital investment and equipment would be a useful next step if the OPGEE model is modified to include embodied energy. The inclusion of embodied energy in steel and cement consumed would reduce these ERRs by an unknown amount. Recent work by Beath et al. [38] suggests that steel consumption (the largest embodied energy impact) ranges from 0.34 kg/bbl to 6.16 kg/bbl. Given energy intensity of steel production from the GREET model (46.5 MJ/kg) and a default energy density per bbl of crude oil (6100 MJ LHV per bbl), this embodied energy will equal 0.3% to 4.7% of the energy content of crude. At the high end, this could result in a ceiling on EROI from crude oil production at a ratio of ≈20:1. Further work would be needed to examine in detail the impacts of embodied energy on NERs.

Another area for fruitful future work would be to use the OPGEE model with detailed field level data collected over many years to determine how oil depletion affects the EROI for oil operations over time. This is not possible to do with the current dataset, which collected a “snapshot” of data for a large number of fields, rather than a time series analysis for a smaller number of oil fields.

Another fruitful avenue for exploration would be to combine refining and upstream energy use into a “well-to-pump” ERR metric. As hydrocarbon resources diverge and various unconventional oils become more prevalent, including refinery energy use would give a more consistent comparison between resources that require on-site upgrading or other pre-processing that might affect refinery energy use. This would likely be achieved using the new open-source refinery model called PRELIM [39]. Recent work on emissions joined the OPGEE and PRELIM models with success [26], and this could be carried over into EROI analysis as well. As a first order approximation, the impact of expanding the system boundary to a “well-to-pump” boundary would add 5–15% of the energy content of the crude as refinery energy consumption [29]. This would greatly reduce NER metrics, introducing a ceiling on NER values set by the large refinery energy consumption. Depending on how refinery energy needs are met (e.g., internally through coke and process gas consumption or externally through purchased natural gas), this boundary expansion could similarly affect resulting EER ratios.

Finally, it is important to recognize that not all MJs are valued equally by various economies into which they are fed. Thus, an asymmetry between the market price of energy produced and energy consumed in the course of production can cause investment and production decisions to diverge from those that would result if energy return metrics such as EROI or NER were used to drive investment decisions.

## Supporting Information

S1 Worksheet Input data for all examined oilfields. (XLSX)
